# GNB1 Encephalopathy: Clinical Case Report and Literature Review

**DOI:** 10.3390/medicina60040589

**Published:** 2024-04-01

**Authors:** Matas Nasvytis, Julija Čiauškaitė, Giedrė Jurkevičienė

**Affiliations:** 1 Independent Researcher, 50234 Kaunas, Lithuania; 2 Department of Neurology, Medical Academy, Lithuanian University of Health Sciences, 44307 Kaunas, Lithuania; julija.ciauskaite@lsmu.lt (J.Č.); giedre.jurkeviciene@lsmu.lt (G.J.)

**Keywords:** GNB1, GNB1 encephalopathy, hereditary encephalopathy, hereditary neurodevelopmental delay, hereditary dystonia

## Abstract

GNB1 encephalopathy is a rare genetic disease caused by pathogenic variants in the G Protein Subunit Beta 1 (GNB1) gene, with only around 68 cases documented worldwide. Although most cases had been caused by de novo germline mutations, in this case, the pathogenic variant was inherited from patient’s mother, indicating an autosomal dominant inheritance pattern. The patient presented at 25 years of age with mild developmental delay and cognitive impairment, prominent generalized dystonia, and horizontal nystagmus which are all characterizing symptoms of GNB1 encephalopathy. Electroencephalography (EEG) showed no epileptiform patterns, and magnetic resonance imaging (MRI) revealed hypointensities in globus pallidus and dentate nucleus areas. The main theory for GNB1 encephalopathy pathogenesis is neuronal hyperexcitability caused by impaired ion channel regulation. Due to low specificity of symptoms, diagnosis relies on genetic testing. As there are no standardized GNB1 encephalopathy treatment guidelines, evaluation of different treatment options is based on anecdotal cases. Reviewing different treatment options, deep brain stimulation and intrathecal baclofen pump, as well as some other medications still in preclinical trials, seem to be the most promising.

## 1. Introduction

GNB1 encephalopathy (GNB1-E) is a rare genetic disease caused by pathogenic variants in the G Protein Subunit Beta 1 (GNB1) gene. Only around 68 cases of this disease have been reported to this day worldwide [1,2,3,4]. Characterizing symptoms for GNB1-E are developmental delay, intellectual disability, and various neurological symptoms. In this article, we report the first case of GNB1-E in Lithuania as well as review the literature related to this disease.

## 2. Materials and Methods

Genetic testing was performed using next-generation sequencing methods by a private company (CeGaT, Tübingen, Germany). The accession number for the patient’s DNA sequence is RCV001254075.2. Testing results were evaluated according to data of ClinVar, dbSNP, Franklin, and Varsome databases. Pathogenicity of revealed variants was evaluated in accordance with American College of Medical Genetics and Genomics (ACMG) guidelines.

Relevant articles selected for literature review were found in electronic National Institutes of Health (NIH) databases using keywords GNB1, GNB1 encephalopathy, GNB1 variants, dystonia, inherited neurologic disorders, gene therapy, and gene therapy in rare neurologic disorders.

## 3. Clinical Case

A 25-year-old female patient presented with impaired speech and gait, tightness in limb muscles, and involuntary jerky movements, impacting her daily life. These symptoms had been present since she was 16 years old. Her medical history indicated delayed speech development, leading to logotherapy. In school, she participated in a special education program but had to discontinue after completing the seventh grade due to the challenges posed by her involuntary movements and speech impairment.

**Scheme 1:**
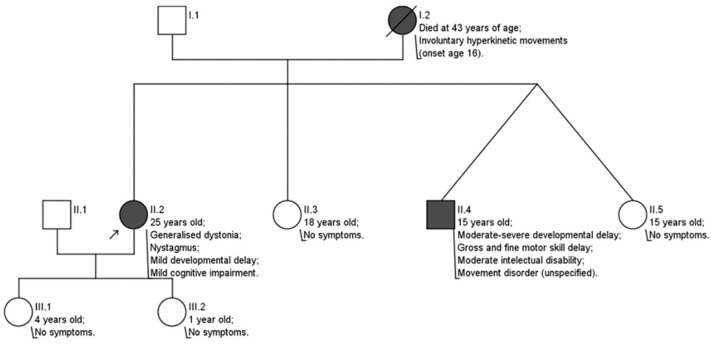
Patient’s pedigree. Note that only the patient herself was genetically tested and clinically evaluated. Information about other family members was provided by the patient.

The patient’s family history revealed that her mother, who passed away at the age of 43 for unknown reasons, experienced similar symptoms starting at age 16. No earlier generations of relatives exhibited similar symptoms. The patient’s 15-year-old brother displayed more severe symptoms, while her two sisters (ages 15 and 18) and two daughters (ages 1 and 4) showed no signs (Scheme 1). Genetic testing and genetic counseling were not pursued for any of the patient’s relatives due to their refusal to participate.

During the neurological examination in the autumn of 2023, the patient exhibited signs of generalized dystonia: dystonic facial expressions, cervical dystonia of the laterocaput type, and there were involuntary limb movements present along with abnormal spasmodic posturing. Her speech was dysarthric and challenging to understand. Anisocoria was observed (left pupil larger than right), with normal pupillary light reflex. Voluntary eye movements were unaffected, but there was horizontal III° nystagmus. Swallowing, muscle strength, sensation, and reflexes were normal. Coordination assessment was challenging due to prominent dystonia. Despite impaired gait, the patient could walk unaided for over 500 m.

Genetic testing performed in 2021 revealed a heterozygous likely pathogenic variant (NM_002074.5(GNB1):c.[226G>A];[226=]) in the 6th exon of the GNB1 gene, confirming a diagnosis of GNB1-E. This variant altered the structure of the guanine nucleotide-binding protein (G protein) β subunit NP_002065.1:p.[(Asp76Asn)];[(Asp=)]. No genetic testing on the patient’s relatives was conducted.

**Figure 1:**
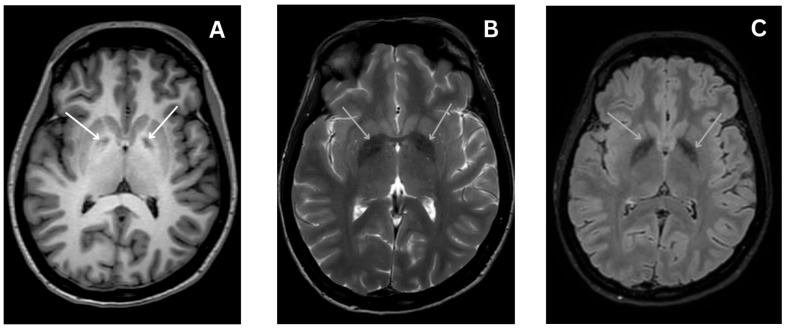
MRI scan axial images show symmetrical bilateral low signal intensity lesions in globus pallidus areas (marked by arrows), best visible in T2-weighted and FLAIR images (**A**)—T1-weighted image; (**B**)—T2-weighted image; (**C**)—FLAIR image).

**Figure 2:**
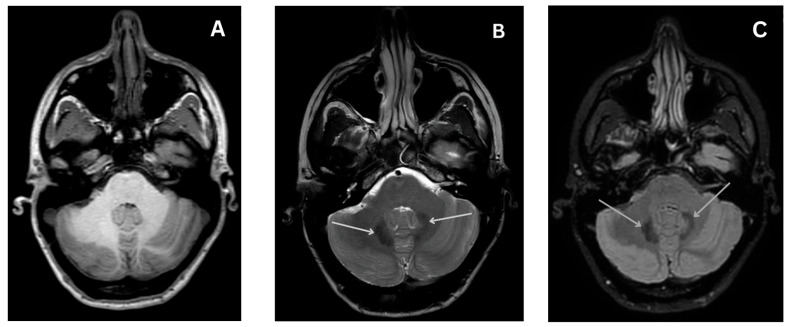
Bilateral low signal intensity lesions in dentate nucleus areas (marked by arrows) visible in T2-weighted (**B**) and FLAIR (**C**) axial images, but not in T1-weighted (**A**) axial image.

In 2020, magnetic resonance imaging (MRI) scans showed T2W/FLAIR hypointensities in the globus pallidus areas bilaterally, consistent with calcification observed in a 2013 computed tomography (CT) scan. Subsequent MRIs in 2024 indicated slightly larger in size hypointensities in the same region, along with new low-signal-intensity abnormalities in the cerebellar dentate nucleus areas (Figure 1 and Figure 2).

Assessments of wakefulness and sleep electroencephalography (EEG) which was performed during planned hospitalization in January 2024 were challenging due to movement artifacts; however, no epileptiform activity was detected. During the same hospitalization, cognitive evaluations using the Mini-Mental State Examination (MMSE) and Montreal Cognitive Assessment (MoCA) scales revealed mild cognitive impairment, with scores of 22 + 2/30 and 16 + 5/30, respectively. The patient also reported high levels of subjective depression (Patient Health Questionnaire-9 (PHQ-9) = 15) and anxiety (Generalized Anxiety Disorder-7 questionnaire (GAD-7) = 16). The patient received consultation from a psychiatrist and was diagnosed with clinical depression. She received treatment with sertraline.

Levodopa treatment was poorly tolerated. Oral administration of baclofen, up to 5 mg three times daily, induced drowsiness without symptom relief and was discontinued after one month. Although clonazepam improved insomnia, it had no impact on dystonia.

Following multidisciplinary team deliberations during the most current hospitalization in January 2024, deep brain stimulation (DBS) was chosen as the next therapeutic intervention.

## 4. Literature Review

### 4.1. Pathogenesis

G protein-coupled receptors (GPCRs) on cell surfaces detect signaling, which is transduced to inner cell structures via G-proteins. These proteins consist of α, β, and γ subunits, with the G-protein β subunit encoded by the GNB1 gene. While subunits are bound together and the α-subunit is bound to a guanosine diphosphate (GDP) molecule, the active sites of the Gαβγ trimer remain inside the protein complex structure, rendering it inactive [5,6,7,8]. Upon GPCR activation, the trimer binds to the receptor’s intracellular region, leading to GDP–guanosine triphosphate (GTP) exchange, dissociation into Gα-GTP and Gβγ complexes, and interaction with intracellular machinery for signal transduction [5,7]. In neurons, Gβγ not only activates kinase-complexes but also directly regulates ion channel activity, including various potassium, calcium, and, most importantly, G protein-coupled inwardly rectifying potassium channels (GIRKs) responsible for hyperpolarization and neuronal excitability [5,9,10].

GNB1-E is usually caused by de novo germline mutations. During the studies of structural changes in Gβ structure caused by pathogenic GNB1 gene variants, Lohman et al. identified several possible mechanisms of signaling disruption: (1) altered Gβ structure leads to unstable Gβγ complex (loss of function—LoF); (2) Gβγ binding to Gα is disrupted, resulting in constantly active Gβγ complex (gain of function—GoF); (3) Gαβγ trimer binding to GPCR is disrupted (GoF) [8].

Petrovski et al. found that 9/13 GNB1-E cases were caused by mutations in exon 6, affecting a narrow amino acid region (Asp76—Ile80) responsible for interaction between Gβγ and Gα. These mutations lead to a constantly active Gβγ complex, impacting Gβγ function in calcium channel inhibition and potassium channel activation [6,11].

Recent studies on mutations in Lys78 and Ile80 positions revealed that the same region of Gβ interacts with both GIRK channels and Gα subunit [9,12]. Reddy et al. demonstrated the direct impact of Lys78 and Ile80 mutations on Gβγ and GIRK channel interaction. Lys78Arg mutation resulted in partial LoF in one of the GIRK clones (GIRK 1/2), at the same time increasing expression of GIRK coding genes, leading to overall GoF in GIRKs. Ile80 mutations caused complete LoF in GIRK channels [9]. Similar results were observed in a study of laboratory mice during which Lys78Arg mutation caused GIRK channel overactivation that resulted in neuronal hyperexcitability in vitro and seizures in vivo, both of which were abolished by GIRK channels inhibitor ethosuximide [12]. Hyperexcitability caused by GIRK activation may sound counterintuitive; however, the leading theory at this time is that Lys78Arg mutation causes hyperactivation of GIRK channels preferentially in inhibitory neurons. As a result, firing of inhibitory neurons is diminished, leading to epileptogenic hyperexcitability of the whole network [12].

### 4.2. Clinical Manifestations

GNB1-E is characterized by moderate to severe developmental delay and/or intellectual disability coupled with at least one of the following neurological symptoms: muscle tone, movement or eye movement disorders, epilepsy, or sensory impairment [13].

#### 4.2.1. Developmental Delay (DD)

There are no data on GNB1-E affecting pregnancy or childbirth [14,15,16]. During the neonatal period, the only symptom is feeding difficulties [3,14]; however, later, DD becomes prominent [3,13,15,16,17]. Moderate to severe global neurodevelopmental delay was documented in almost all GNB1-E cases [3,6,8,13,14]. Most of the patients were unable to walk independently [13,14] or started walking late. All walking patients exhibited prominent gait disorders and frequent falls [3,13,14,16]. In some cases, patients developed hemiplegia [6], severe dyskinetic quadriplegia [6,17], and spastic diplegia [13,17]. According to various authors, 40% to 72% of the patients were nonverbal [6,13,14]. Regarding speaking patients, expressive language was more affected than receptive, and patients had limited vocabulary and were dysarthric [3,14,15]. Growth delay in early years due to inability to feed was documented in 38% to 50% cases [3,6,8,13,14,17]; however, most patients caught up in later years [13,14]. Developmental regression was very rare (3/57) [13].

#### 4.2.2. Intellectual Disability (ID)

ID is present in 74% [13] to 87% [8] cases and is of variating severity [3,8,13,15,16,17].

#### 4.2.3. Neurological Symptoms

- Muscle tone disorders. Hypotonia is the most common clinical finding in infancy ranging from mild to severe, generalized forms that result in hypotonic quadriplegia and oropharyngeal dysphagia [6,8,14,16,17]. In rare cases, hypotonia persists with age [6]; however, usually, it transforms into spastic hypertonia. Some studies highlight the pattern of high limb and low axial muscle tone [6,13,14,16,17].
- Movement disorders. The most commonly documented movement disorder is dystonia, varying from mild dystonic positioning of the fingers to generalized dystonia with age of onset as early as 2 years old and as late as 16 years old [3,8,13,14,15,16,17]. In some cases, myoclonus [3,15,16] or intermittent status dystonicus [15,17] were described. Other findings include tics [6], ataxia, or chorea [6,8,13].
- Epilepsy. Epilepsy manifests in some, but not all, cases (10/13 [6] and 6/18 [14]). Seizures can be focal or generalized, and several cases of status epilepticus were also documented [6,14]. Seizure morphology varies; tonic, atonic, myoclonic, tonic-clonic, absence seizures, and infantile spasms were documented [6,8,13,14,17]. GNB1 gene variants are also associated with West syndrome [14,17].
- Eye movement disorders. The most common eye movement disorder is nystagmus (36–46%). It can be horizontal, vertical, rotatory, or of alternating direction [6,13,14,16]. Strabismus, ophthalmoplegia, gaze deviation, upward gaze palsy, and slow ocular pursuit were also documented [3,6,8,13,14,15,17].
- Visual impairment. In 11% of cases, cortical visual impairment, cortical blindness, or optic nerve atrophy were diagnosed [6,13,14,17]. Additionally, one case of rod-cone dystrophy was reported [18].
- Hearing impairment. In rare cases, unilateral or bilateral sensorineural hearing loss was present [6,13,14].

#### 4.2.4. Other Symptoms

Other symptoms include autism spectrum disorders, attention deficit and hyperactivity disorder (ADHD), gastrointestinal tract disturbances, genitourinary abnormalities (only in males), cardiovascular abnormalities, craniofacial malformations, cutaneous mastocytosis, and rare endocrine system disorders [6,13,14,15,16].

### 4.3. Prognosis

Given that the overwhelming majority of reported GNB1-E cases in the literature pertain to children or young adults, the life expectancy remains undetermined [13]. The cause of death in GNB1-E patients is largely not known, as is the case of the patient’s mother [13,14]. The few cases of adult GNB1-E patients reported in the scientific literature in addition to our patient’s and her mother’s cases prove that survival into adulthood is feasible [3,4,6,13]. These data, coupled with the absence of congenital anomalies linked to heightened morbidity and mortality, leads us to speculate about a favorable prognosis with appropriate management and support [13].

### 4.4. Diagnostics

#### 4.4.1. Genetic Testing

GNB1-E diagnosis can only be established via genetic testing, starting with chromosomal microarray analysis (CMA) for large chromosomal abnormalities. If CMA is not diagnostic, next-generation sequencing methods, like multigene panels or comprehensive genomic testing, are recommended. A pathogenic or likely pathogenic GNB1 variant confirms the diagnosis, with both terms considered synonymous [13,18].

#### 4.4.2. Laboratory Testing

Despite various markers being evaluated in GNB1-E patients, including complete blood count, serum copper, serum ceruloplasmin, serum organic, and amino acid levels, as well as neurotransmitter levels in cerebrospinal fluid, no significant changes have been identified, and no associated laboratory markers currently exist [8,15].

#### 4.4.3. Electroencephalography

Individuals diagnosed with GNB1-E, even without epilepsy symptoms, are advised to undergo sleep and wakefulness EEG in order to record and characterize any abnormal episodes or subclinical seizures [13]. Epileptogenic forms of GNB1-E manifest with EEG abnormalities such as spike and wave complexes, polyspikes, slow waves, hypsarrhythmia, and patterns consistent with infantile spasms [6,14,17]. Studies on laboratory mice with Lys78Arg GNB1 mutation mirrored these findings, confirming the role of pathogenic GNB1 variants in epileptogenic activity [12].

#### 4.4.4. Magnetic Resonance Imaging

While not present in every case, MRI is recommended for all GNB1-E patients [13]. Potential abnormalities include delayed myelination, corpus callosum abnormalities, cerebral volume loss, ventriculomegaly and increased fluid in subarachnoid spaces or central canal, bilateral polymicrogyria, cerebellar hypoplasia, diffuse, nonspecific white matter abnormalities, especially near ventricles, and caudate nucleus abnormalities [6,8,13,14].

### 4.5. Treatment

Currently, no etiologic treatment exists for GNB1-E, and symptom management remains the primary focus [13].

Given the variable degree of DD and ID in GNB1-E, early multidisciplinary interventions are crucial. Infants may benefit from feeding therapy, physiotherapy, occupational therapy, and consultation with sensory impairment specialists. For preschool and school-age children, an individualized education plan addressing sensory impairment and developmental delays is essential. Psychotherapy, continued physical therapy, and walking aids play crucial roles [13].

Standard antiepileptic drugs are used to treat epilepsy caused by GNB1-E. While no specific drug preference is confirmed by clinical trials, a study on a laboratory mouse model demonstrated effective treatment of epileptiform activity with ethosuximide [12]. In one case, a patient experiencing infantile spasms at four months of age achieved spasm remission following adrenocorticotropic hormone (ACTH) therapy. However, a relapse occurred six months later [17]. In a different case, a patient diagnosed with West syndrome saw a recurrence of spasms after the initial ACTH therapy, but with repeated ACTH therapy, the spasms eventually ceased, and subsequent EEG monitoring revealed no epileptiform activity [17].

Standard treatments for dystonia and movement disorders are recommended in the literature, including anticholinergic drugs, dopamine analogues, agonists, antagonists, depleters, benzodiazepines, or baclofen [13,19]. DBS is the preferred method of neuromodulation. Due to the rarity of the disease, there are no standardized treatment guidelines, and treatment is individualized, with variable responses to medications. Trihexyphenidyl showed limited success, reducing myoclonus and causing agitation [15], levodopa worsened dystonia [15,16], and clonazepam had no significant effect [15]. Some cases were treated with risperidone, dantrolene, sodium hydrate, and phenobarbital, but success was limited [17]. Clobazam, haloperidol, valproate, methylphenidate, and olanzapine either worsened or had no effect on symptoms [15]. Despite it being one of the choices in dystonia treatment, no attempts of administering baclofen either orally or intrathecally were reported. In one case, bilateral globus pallidus internus DBS improved myoclonus instantly and dystonia temporarily [15].

Physiotherapy and occupational therapy are essential to prevent contractures, falls, and complications caused by spasticity [13,14].

Disorders in other systems caused by pathogenic GNB1 gene variants should be managed by appropriate specialists [13].

## 5. Discussion

GNB1-E is primarily caused by de novo germline mutations, but this case stands out as the patient inherited the pathogenic GNB1 variant from her mother. Up to this day, only five cases of GNB-1 gene variant inheritance have been reported, with two being unknown mode and three being autosomal dominant mode of inheritance [8]. In this case, familial history data are consistent with autosomal dominant mode of inheritance.

Although the patient’s symptoms are characteristic for GNB1 encephalopathy, they are not specific for this disease, especially as the patient exhibits a mild phenotype. Low specificity of clinical manifestation necessitates genetic testing for establishing diagnosis. Next-generation sequencing (NGS), particularly multigene panels, aids in diagnosis by selecting panels relevant to the most prominent symptoms.

The reluctance of the patient’s relatives to participate in genetic testing raises the issue of genetic counselling. Given that GNB1-E manifests as a monogenic disorder inherited in an autosomal dominant manner, the family members of the patient stand to benefit from comprehensive genetic testing and counseling [13]. This is particularly pertinent as the pathogenic GNB1 variant in this instance appears to be inherited rather than arising from de novo mutation. Even in scenarios where pathogenic GNB1 variants are detected only in the proband without identification in either parent, the risk to siblings exceeds that of the general population due to the potential presence of parental germline mosaicism. Hence, genetic counseling is strongly recommended [13]. Nonetheless, it is imperative to uphold the fundamental principles of genetic testing, which include both the patient’s and their family’s autonomy and the right to not know. It is essential to ensure that members of the patient’s family are not coerced into undergoing genetic testing [20]. In this context, the decision not to undergo testing for the patient’s siblings was made by their father, considering their minor status at the time. Upon reaching adulthood and achieving autonomy, they should be informed of the available genetic counseling options, including prenatal and preimplantation testing, empowering them to make well-informed reproductive decisions [13,20].

In this case, the likely pathogenic GNB1 variant is missense mutation located in the sixth exon replacing guanine with adenine in the 226th position. This results in replacing aspartate with asparagine in the 76th position of the Gβ amino acid chain. Although this exact mutation has not been described previously, the 76^th^ position belongs to the aforementioned narrow G-protein region (Asp76—Ile80) responsible for binding between Gβ and Gα and Gβγ dimer interactions with GIRK channels [6,9,12]. Although GIRK overactivation results in epileptogenic activity, some other cases of mutations in the 76^th^ position also had no epileptogenic manifestation [6,14]. This necessitates further studies on the molecular structures of different Gβ subunit protein residues to better explain molecular mechanisms in play.

Current research explores ion channel regulation disturbances linked to GNB1 gene variants resulting in neuronal hyperexcitability and epileptiform patterns [9,12]. However, the origin of hyperkinesia, hypertonia, and dystonia is fundamentally similar—neuronal hyperexcitability. In a laboratory mouse model study, Colombo et al. researched epilepsy caused by GNB1 mutation Lys78Arg, which is close to the patient’s mutation (Asp76Asn), and reached promising results in using the GIRK channel blocker ethosuximide [12]. Reddy et al. demonstrated that mutations in the Ile80 position cause GIRK channel suppression, also resulting in neuronal hyperexcitability, and that those patients may benefit from GIRK openers VU0529331 and ML297 [9]. However, the effectiveness and safety of these medications still need to be proven in clinical trials.

Another treatment option to consider is intrathecal baclofen pump. In our case, baclofen administered orally in doses of 5 mg thrice daily did not improve the patient’s symptoms and caused drowsiness. Transient sedation stands out as a prevalent adverse reaction to oral baclofen and, thus, stands as one of the main limiting factors in reaching effective dosage [21]. It is plausible that the lack of symptom improvement in this instance may be attributed to insufficient dosage due to intolerable side effects. However, response to oral baclofen is a poor predictor of intrathecal baclofen efficacy [19,21].

Several different MRI scans showed hypointense signal lesions in globus pallidus areas bilaterally. In one clinical case, DBS in globus pallidus internus areas significantly reduced GNB1-E symptoms. The patient received DBS at 15 years of age, when dystonia and myoclonus began interfering with daily activities [15]. The case in mention has many similarities with the case we are reporting; in both cases, the GNB1 variant phenotype is mild and age of onset for dystonia and myoclonus is similar. Based on this clinical practice and the fact that DBS is the main method of neuromodulation in dystonia treatment [19], DBS has a lot of potential in this specific case.

With the advent of the gene therapy era, new prospects for addressing genetic disorders, particularly monogenetic ones, have emerged. Recent FDA endorsements of gene therapy for treating spinal muscular dystrophy (SMA) and RPE65 mutation-associated retinal dystrophy offer the promise that a multitude of rare neurological disorders may soon find therapeutic avenues through gene therapy [22]. Presently, clinical trials are underway for addressing conditions such as Tay–Sachs disease (TSD), Sandhoff disease (SD), Huntington’s disease, Canavan disease, Batten disease, Krabbe disease, Niemann–Pick disease, multiple system atrophy, amyotrophic lateral sclerosis, metachromatic leukodystrophy, X-linked adrenoleukodystrophy, phenylketonuria, and gangliosidoses [23]. The rapid evolution of gene therapy treatments fosters optimism regarding potential treatment options for GNB1-E, even in the absence of preclinical trials. However, the main obstacle in this trajectory remains the extreme rarity of the disease, posing challenges in recruiting a sufficient number of patients for future clinical trials, and the inadequate financial incentive for pharmaceutical companies to pursue this avenue of research due to the low patient count. Another obstacle in developing gene therapy for GNB1-E could be the prevalence and vital role of G-proteins across organ systems, necessitating broad cellular transduction similar to gene therapies being formulated for TSD and SD. Another promising notion—the advancement of CRISPR/Cas9 technology presents the theoretical possibility of rectifications as minute as a single nucleotide, potentially paving the way for personalized gene therapies targeting specific mutations [22,23].

## 6. Conclusions

This case of GNB1-E is notable for the inherited GNB1 variant, contrasting with the usually reported de novo germline mutations. GNB1-E is an exceptionally rare and heterogenous disease, lacking standardized treatment guidelines; thus, treatment selection is often based on anecdotal evidence or predominant symptoms. Given the main clinical manifestation of dystonia in this case and the ineffectiveness of various oral medications, deep brain stimulation (DBS) was chosen. Other promising treatment options described in the scientific literature include intrathecal baclofen pump and other medications undergoing preclinical trials. We are optimistic that advancements in genetic diagnostics will lead to improved detection rates of GNB1 encephalopathy, fostering a deeper understanding of the disease and the development of more effective treatment strategies. Gene therapy emerges as a highly promising avenue, as an increased number of diagnosed patients would not only amplify the financial incentive for advancing gene therapy solutions but also streamline the process of recruiting adequate numbers of participants for clinical trials.

## Data Availability

The original contributions presented in the study are included in the article, further inquiries can be directed to the corresponding author.
